# Biomarkers and perfusion – training-induced changes after stroke (BAPTISe): protocol of an observational study accompanying a randomized controlled trial

**DOI:** 10.1186/1471-2377-13-197

**Published:** 2013-12-11

**Authors:** Alexander H Nave, Jan M Kröber, Peter Brunecker, Jochen B Fiebach, Jonathan List, Ulrike Grittner, Matthias Endres, Andreas Meisel, Agnes Flöel, Martin Ebinger

**Affiliations:** 1 Center for Stroke Research Berlin (CSB), Charité - Universitätsmedizin Berlin, Charitéplatz 1, 10117 Berlin, Germany; 2 Department of Neurology, Charité - Universätsmedizin Berlin, Berlin, Germany; 3 NeuroCure Clinical Research Center, Charité - Universätsmedizin Berlin, Berlin, Germany; 4 Deutsches Zentrum für Herz-Kreislauf-Forschung (DZHK), Standort Berlin, Germany; 5 Department for Biostatistics and Clinical Epidemiology, Charité - Universätsmedizin Berlin, Berlin, Germany

**Keywords:** Physical training, Exercise, Subacute stroke, Ischemic stroke, Cerebral perfusion, Biomarkers, Vessel size imaging, Neovascularization, MRI, Rehabilitation

## Abstract

**Background:**

Physical activity is believed to exert a beneficial effect on functional and cognitive rehabilitation of patients with stroke. Although studies have addressed the impact of physical exercise in cerebrovascular prevention and rehabilitation, the underlying mechanisms leading to improvement are poorly understood. Training-induced increase of cerebral perfusion is a possible mediating mechanism. Our exploratory study aims to investigate training-induced changes in blood biomarker levels and magnetic resonance imaging in patients with subacute ischemic stroke.

**Methods/design:**

This biomarker-driven study uses an observational design to examine a subgroup of patients in the randomized, controlled PHYS-STROKE trial. In PHYS-STROKE, 215 patients with subacute stroke (hemorrhagic and ischemic) receive either 4 weeks of physical training (aerobic training, 5 times a week, for 50 minutes) or 4 weeks of relaxation sessions (5 times a week, for 50 minutes). A convenience sample of 100 of these patients with ischemic stroke will be included in BAPTISe and will receive magnetic resonance imaging (MRI) scans and an additional blood draw before and after the PHYS-STROKE intervention. Imaging scans will address parameters of cerebral perfusion, vessel size imaging, and microvessel density (the Q factor) to estimate the degree of neovascularization in the brain. Blood tests will determine several parameters of immunity, inflammation, endothelial function, and lipometabolism. Primary objective of this study is to evaluate differential changes in MRI and blood-derived biomarkers between groups. Other endpoints are next cerebrovascular events and functional status of the patient after the intervention and after 3 months assessed by functional scores, in particular walking speed and Barthel index (co-primary endpoints of PHYS-STROKE). Additionally, we will assess the association between functional outcomes and biomarkers including imaging results. For all endpoints we will compare changes between patients who received physical fitness training and patients who had relaxation sessions.

**Discussion:**

This exploratory study will be the first to investigate the effects of physical fitness training in patients with ischemic stroke on MRI-based cerebral perfusion, pertinent blood biomarker levels, and functional outcome. The study may have an impact on current patient rehabilitation strategies and reveal important information about the roles of MRI and blood-derived biomarkers in ischemic stroke.

**Trial registration:**

NCT01954797.

## Background

The importance of physical exercise in cardiovascular disease prevention is evident [1,2]. Physical fitness can mitigate clinically relevant risk factors including hypertension, diabetes mellitus, or dyslipidemia [3]. Physical exercise may also be favorable in primary and secondary prevention of cerebrovascular diseases and cognitive impairment [4]. Large prospective cohort studies have highlighted the association of midlife fitness levels and lower hazard ratios of developing later-life dementia; it has been shown that physical activity has beneficial effects on structural brain integrity [5-7]. Aerobic fitness training was indicated to have a positive impact on the functional outcome of patients with stroke [8,9]. However, the underlying mechanisms leading to improved clinical rehabilitation are not well understood and there is need for further studies adding information to the growing evidence [4,8].

The change of cerebral perfusion following physical activity could be one possible mechanism to explain the observed clinical improvement in patients. Impaired cerebrovascular reactivity has been associated with various conditions including age, white matter lesion pathology, cognitive impairment, and risk of stroke [10-12]. Resting cerebral blood flow declines with age [13] and endurance exercise-trained men show increased cerebral blood flow velocity compared to sedentary men of same age [14,15]. More recent studies have underscored the positive effects of physical exercise in healthy individuals and chronic stroke patients; they have linked the effects to improved cerebral perfusion and autoregulation [16,17]. In addition, current magnetic resonance imaging (MRI) scans using vessel size imaging enable in vivo assessment of cerebral perfusion, microvascular morphology, and cerebral angiogenesis; hence providing promising information about vascular remodeling, neural plasticity, and cerebral (neo)vascularization after stroke [18,19].

Preclinical studies have shown beneficial effects of physical exercise on long-term stroke outcome in rodents. These outcomes were associated with increased basal cerebral blood flow and improved endothelial function as well as angiogenesis [20-22]. Other animal studies have reported that certain serum biomarkers strongly correlate with functional outcome after stroke [23,24]. In humans, presence of different blood biomarkers including stress and immune markers are known to have a negative prognostic value on the outcome of patients with ischemic stroke [25-27]. Investigations on the influence of physical activation and exercise on immunity revealed conflicting results. Whether or not pertinent blood biomarker levels can be positively influenced by physical exercise post stroke has not been investigated so far.

A better understanding of physical training-induced changes in cerebral perfusion and cerebral microvasculature in correlation with alterations of specific serum biomarker levels and functional status of patients would have a considerable effect on stroke prevention, rehabilitation and could lead to new therapeutic strategies.

## Methods/design

### Setting

“Biomarkers And Perfusion – Training-Induced changes after Stroke” (NCT01954797) is a prospective, endpoint-blinded observational study accompanying a randomized, controlled trial called Physical Fitness Training in Subacute Stroke (NCT01953549). Out of all PHYS-STROKE participants, only patients with ischemic stroke are eligible for BAPTISe. Patients will be recruited at six different participating rehabilitation sites in the Berlin area. Recruitment will start in October 2013 and will continue for 30 months in total. Follow-up visits are planned at 3 and 6 months post stroke. The duration of the study will be 36 months in total. Research is carried out in compliance with the Helsinki Declaration and received ethical approval of the local authorities (Ethics Commission of the Charité, EA1/137/13).

Although this study is accompanying a randomized controlled trial we consider it to be similar to a prospective observational study where the physical training is the exposure. Reasons for this are:

The PHYS-STROKE trial is designed for different outcomes (functional outcomes) than the BAPTISe study.

Not all patients from PHYS-STROKE will be included in BAPTISe. We will use convenience sampling from participants in the trial. In sensitivity analysis we will test whether the subgroup of patients in BAPTISe differs from those patients in PHYS-STROKE eligible for BAPTISe but not included in BAPTISe.

The analysis of the endpoints (MRI and blood biomarkers) is exploratory insofar as this study tries to identify potential candidates of MRI measures and blood biomarkers that are associated with physical training in subacute ischemic stroke patients after adjustment for possible confounders.

We aim to determine specific changes in the blood and the cerebral vasculature of patients with subacute ischemic stroke who perform 4 weeks of aerobic fitness training in comparison to patients who receive 4 weeks of relaxation sessions. Serum levels of multiple blood biomarkers including parameters of immunity, inflammation, endothelial function, and lipometabolism will be assessed from all patients enrolled in PHYS-STROKE. Blood draws will also be performed at follow-up visits after 3 and 6 months (as part of the PHYS-STROKE trial). Blood-derived biomarkers assessed are listed in Table 1. Patients who also enrolled in BAPTISe will have an MRI scan and an additional blood draw for the assessment of certain markers of endothelial function in the vicinity of the MR scanner before and after the intervention. Using MRI we will assess various parameters of cerebral perfusion measuring cerebral blood flow (CBF) and cerebral blood volume (CBV), and try to determine the change in cerebral neovascularization by computation of the Q factor using vessel size imaging. The Q factor is a calculated ratio that correlates with the degree of microvessel density (MVD) [19,28]. Additionally, a MRI-based determination of the intra-abdominal fat mass will be performed on all patients with the second MRI scan after intervention. We will furthermore analyze the association of observed changes in blood biomarker levels with MRI findings and functional outcomes of patients.

**Table 1:** List of tested blood biomarkers

**Blood biomarkers**	**Time points**			
	**Baseline (t1)**	**t1+ 4 weeks (t2)**	**3 months post stroke (t3)**	**6 months post stroke (t4)**
Glucose	X	X	X	X
HbA1c	X	X	X	X
INR	X			
PTT	X			
Fibrinogen	X	X	X	X
LDL	X	X	X	X
HDL	X	X	X	X
Triglycerides	X	X	X	X
Lipoprotein a	X	X	X	X
Full blood count	X	X	X	X
Transaminases	X	X	X	X
Creatinine	X	X	X	X
Cortisol	X	X	X	X
Insulin	X	X	X	X
TSH	X		X	
fT3	(X)		(X)	
fT4	(X)		(X)	
FSH				X
LH				X
Testosterone				X
Estradiol				X
hs-CRP	X	X	X	X
TNF-alpha	X	X	X	X
IL-6	X	X	X	X
HLA-DR	X		X	X
Lymphocyte subpopulations	X		X	X
LBP	X		X	X
MBL	X		X	X
Copeptin	X		X	X
proANP	X		X	X
IL-1b	RS	RS	RS	RS
BDNF	RS	RS	RS	RS
IGF-1	RS	RS	RS	RS
IGFBP-3	RS	RS	RS	RS
G-CSF	RS	RS	RS	RS
NGF	RS	RS	RS	RS
Endothelial progenitor cells	RS	RS	RS	RS
Endothelial microparticles	RS	RS	RS	RS
Endoglin	RS	RS	RS	RS
Apelin	RS	RS	RS	RS
Irisin	RS	RS	RS	RS
CNTF	RS	RS	RS	RS
GFAP	RS	RS	RS	RS
VEGF	RS	RS	RS	RS
VEGFR	RS	RS	RS	RS
Other	RS	RS	RS	RS

Spectrum of blood biomarkers assessed in the first 24 patients. Parameters which have proved to be most pertinent will be further evaluated in the following patients of BAPTISe.

X = biomarker detection; (X) = detection if TSH is abnormal; RS = reserve samples for later testing. HbA1c: glycated hemoglobin; INR: international normalized ration; PTT: partial thromboplastin time; LDL: low-density lipoprotein; HDL: high-density lipoprotein; TSH: thyreotropin; fT3: triiodothyronine; fT4: thyroxine; FSH: follicle-stimulating hormone; LH: luteinizing hormone; TNF-alpha: tumor necrosis factor alpha; IL-6: interleukin-6; HLA-DR: human leucocyte antigen-DR; LBP: lipopolysaccharide-binding protein; MBL: mannose-binding lectin; pro-ANP: pro-atrial natriuretic peptide BDNF: brain-derived neurotrophic factor; IGF-1: insulin-like growth factor 1; IGFBP-3: insulin-like growth factor-binding protein 3; G-CSF: granulocyte colony-stimulating factor; CNTF: ciliary neurotrophic factor; GFAP: glial fibrillary acidic protein; VEGF: vascular endothelial growth factor; VEGFR: vascular endothelial growth factor receptor.

### Sample size and power calculation

We chose a two-stage study design. After recruitment of the first 24 patients an interim analysis will be performed (first stage). The two-stage approach will give us the opportunity to generate a more specified hypothesis and evaluate the most pertinent biomarkers in more detail and conduct the study with recruitment of up to 76 remaining patients (second stage).

Based on animal studies reporting a training-induced CBF increase [20] we expect CBF to increase about 30% after the 4-weeks intervention of physical exercise. The PHYS-STROKE trial is aiming to recruit 215 patients. Due to the stricter inclusion criteria of BAPTISe (ischemic stroke, MRI eligibility) we estimate that approximately 50% of all PHYS-STROKE patients can be included in BAPTISe. Including 100 patients and allowing for a 20% drop-out/loss to follow-up rate, the number of patients to be analyzed would be 80. With a sample size of 80 patients (40 in each group) it is possible to detect differential changes between groups with a power of 80% and a significance level alpha of 0.05 if the effect size is 0.64. Since this is an exploratory study no adjustment for multiple testing will be applied.

### Patient population - inclusion and exclusion criteria

Patients (male/female) enrolled in PHYS-STROKE aged 18 years or older with ischemic stroke in the subacute phase (5–45 days after onset) are eligible to participate in BAPTISe. In addition to patients’ ability to perform aerobic exercise, determined by the responsible physician, they must be able to undergo MRI scans. For all inclusion and exclusion criteria please refer to “inclusion and exclusion criteria of BAPTISe” section. Screening, recruitment, baseline assessment, and follow-up visits will take place at the recruiting rehabilitation sites in the Berlin area. MR imaging will take place at the Charité - Universitätsmedizin Berlin.

Inclusion criteria

Age: > 18 years

Diagnosis of subacute ischemic stroke (within 5–45 days after stroke onset) as determined by initial MRI/CT scan of the brain

Cortical, sub-cortical, or brainstem affection

Barthel Index (BI) <65 at inclusion

Able to sit for at least 30 seconds (unsupported or supported, i.e., holding onto supports such as the edge of the bed)

Ability to perform aerobic exercise, determined by responsible physician

Provision of written informed consent

Exclusion criteria

Lacking ability to comply with study requirements

Stroke due to intracranial hemorrhage

Previous subarachnoid hemorrhage or other hemorrhagic stroke

Progressive stroke

Not able to receive magnetic resonance imaging scans, including perfusion imaging

Unable to perform the required exercises due to medical, musculo-skeletal, or neurological problems

Required help of at least 1 person to walk before stroke due to neurological (e.g., advanced Parkinson’s disease, Amyotrophic Lateral Sclerosis, Multiple Sclerosis) or non neurological co-morbidities (e.g. heart failure, orthopedic problems)

Life expectancy < 1 year as determined by responsible physician

Drug or alcohol addiction within the last six months

Significant current psychiatric illness defined as medication-refractory of bipolar affective disorder, psychosis, schizophrenia or suicidality

Current participation in another interventional trial

**Figure 1:**
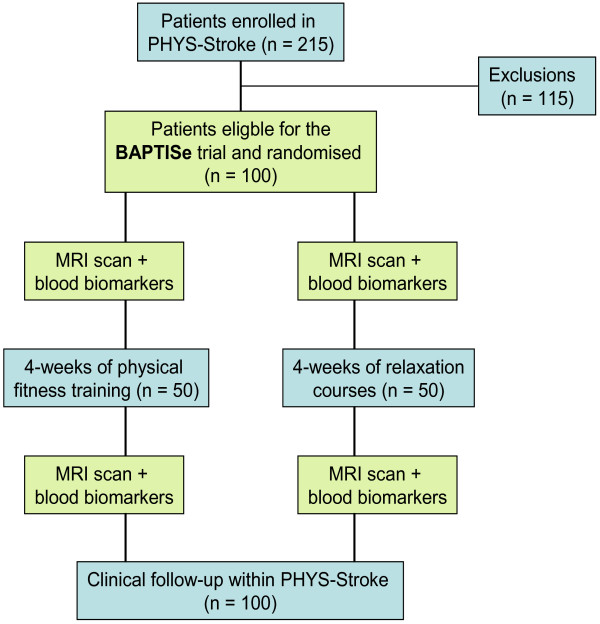
Flow chart of the BAPTISe trial.

We will use convenience sampling of participants in the PHYS-STROKE trial. Informed consent for both studies is obtained simultaneously. After informed consent for PHYS-STROKE and BAPTISe is obtained participating patients will be randomized 1:1 either to the experimental interventional group (physical fitness training) or to the control interventional group (relaxation) of PHYS-STROKE, as shown in the Flow Chart in Figure 1.

BAPTISe examines a subgroup within a randomized controlled trial, PHYS-STROKE (details of the intervention are described elsewhere; NCT01953549). In brief, patients in the interventional arm of the PHYS-STROKE trial will participate in a 4-week physical training course. The physical training includes aerobic training for 50 minutes five times a week. Patients will receive a total of 20 sessions, in addition to standard rehabilitative treatment. Patients in the control arm will take part in relaxation sessions additionally to the standard rehabilitative treatment.

### Observation

All patients enrolled in the PHYS-STROKE trial will have blood draws before and after the intervention, and at follow-up visits at 3 and 6 months post stroke (as part of the PHYS-STROKE trial) to determine biomarkers levels of immunity, inflammation, endothelial function, and lipometabolism, considered relevant in (recurrent) stroke [24,26,29-32]. A list of tested blood-derived biomarkers is provided in Table 1. Patients of both groups (intervention/control) who have also consented to participate in BAPTISe will receive a cerebral MRI scan and an additional blood draw for the assessment of markers of endothelial function in the vicinity of the MR scanner before and after the PHYS-STROKE intervention. The MRI is performed in order to assess the degree of cerebral perfusion changes and changes of the cerebral microvasculature using vessel size imaging – a technique to quantitatively map the cerebral microvascular morphology [28,33]. Various parameters of perfusion and vascularization including cerebral blood flow (CBF), cerebral blood volume (CBV), microvessel density (Q factor), and vessel size index (VSI) will be measured. Previous preclinical and clinical studies have underscored the useful potential of these imaging biomarkers in monitoring post-ischemic brain tissue processes, such as angiogenesis and vascular remodeling [18,19,28,33,34]. Imaging will be performed with a 3 Tesla Siemens TIM Trio at the Department for Neurology, Charité - Universitätsmedizin Berlin. The complete MRI sequence protocol is listed in Table 2.

**Table 2:** MRI sequences

**#**	**Sequence name**	**Details**	**Duration**
(1)	T2	abdominal TSE with water suppression (only 2^nd^ scan)	2:13
1	Localizer	3 × 3	0:11
2	FLAIR	5 mm slice thickness	1:54
3	Resting state	3 × 3 × 4 mm × 150 scans, TR = 2.3 s	6:44
4	DTI	64 directions plus 9× B0, 2.3 mm isovoxel	10:04
5	MPRAGE	1 mm isovoxel	4:26
6	MRA	TOF, 0.5 × 0.5 × 0.7 mm	2:53
7	T1	2D FLASH, flip angles 25°/50°	0:40
8	DWI	6 directions, 5 mm slice thickness	1:30
- contrast application (gadobutrol) -			
9	VSI / PWI	combined SE-GRE, TE = 22 ms/85 ms	2:31
10	T1	2D FLASH, flip angles 25°/50°	0:40

### Outcomes

The exploratory study design of BAPTISe will allow us to generate a specified hypothesis and narrow down the study endpoints after the analysis of data from the first 24 patients. We will analyze the association of primary and secondary endpoints which are specified after the interim analysis with functional outcome, in particular Barthel Index (BI), walking speed (co-primary endpoints of PHYS-STROKE), and modified Rankin Scale (mRS).

For imaging sequences see Table 2, which includes cerebral blood flow (CBF) and cerebral blood volume (CBV), vessel size imaging, and computation of the Q factor to assess cerebral microvessel density (MVD). The assessment of the intra-abdominal fat mass of patients will only be performed with the second MRI scan after intervention. Changes of pertinent blood biomarker levels listed in Table 1 will be correlated with MRI changes, next cerebrovascular events, and functional status directly after the intervention and 3 months post stroke.

#### Data monitoring body

Adverse events emerging in the PHYS-STROKE trial are registered within 24 hours of occurrence and immediately reported to the study physician. Data Safety and Monitoring Board (DSMB) is informed of all serious adverse events and study progress.

### Statistical analysis

Based on feasibility of data collection the final sample size will include up to 80–100 patients.

Differential changes in measures between groups from baseline to follow up (4 weeks, 3 months, and 6 months) will be analyzed separately for every measure using ANCOVA with baseline measures and exposure (physical training) as covariates and follow up measure as outcome.

In further analyses it will be accounted for additional covariates such as age, sex, stroke severity, and diffusion-weighted imaging (DWI) lesion volume. In order to analyze the complete longitudinal course of the data linear mixed effects models will be used for the analysis.

Additionally we will analyze how changes in MRI measures and blood markers are related to each other and to differences in functional outcome, in particular gait speed, Barthel index (co-primary endpoints of PHYS-STROKE) and mRS, after adjustment for treatment exposure using appropriate statistical methods.

### Interim analysis

The aim of the interim analysis of the first 24 recruited patients is to confirm feasibility and update the analysis plan, identifying the most promising primary endpoint and excluding less promising blood markers from further analysis that are not associated with the exposure (physical training) defined by effect sizes for differential changes between groups of less than 0.3.

### Sensitivity analysis

We will compare baseline characteristics (age, sex, stroke severity, DWI lesion size) between those patients included in BAPTISe and eligible patients in PHYS-STROKE who were not included in BAPTISE to test if patients in BAPTISe are comparable to the study population in PHYS-STROKE.

### Study organization and funding

BAPTISe is funded by the Federal Ministry of Education and Research through the grant G.2.15 of the Center for Stroke Research Berlin (CSB).

## Discussion

The goal of this clinical study is to show that physical fitness training of patients in the subacute phase of ischemic stroke leads to changes in imaging and blood-derived biomarkers which correlate with improved functional outcome. The study will provide important information about the roles of imaging and blood biomarkers in subacute stroke rehabilitation. It thus might impact current rehabilitation strategies representing advantageous methods of monitoring future treatment plans that address the promotion of cerebral perfusion and neovascularization after stroke.

### Trial status

Patient recruitment will start in October 2013 and is aimed to continue for 30 months in total. Last follow-up is scheduled for October 2016.

## Abbreviations

BI: Barthel Index; mRS: modified Rankin Scale; Q: quantity of microvessel density; VSI: vessel size index; CBF: cerebral blood flow; CBV: cerebral blood volume; CSB: Center for Stroke Research Berlin; DSMB: Data Safety and Monitoring Board; DWI: diffusion-weighted imaging.

## Competing interests

The authors declare that no conflicts of interest exist.

## Authors’ contributions

AHN prepared the manuscript and has been involved in planning BAPTISe; JMK, JL, AM and MEnd thoroughly revised the manuscript; MEbi supervises and designed BAPTISe and has been involved in drafting and revising the manuscript; UG revised the manuscript and contributed statistical expertise; AF supervises the PHYS-STROKE trial and revised the manuscript; PB and JF are in charge of MR imaging and revised the manuscript. All authors read and approved the final manuscript.

## Pre-publication history

The pre-publication history for this paper can be accessed here:

http://www.biomedcentral.com/1471-2377/13/197/prepub
